# Effect of metformin on sepsis-associated acute lung injury and gut microbiota in aged rats with sepsis

**DOI:** 10.3389/fcimb.2023.1139436

**Published:** 2023-03-09

**Authors:** Youdong Wan, Shuya Wang, Yifan Niu, Boyang Duo, Yinshuang Liu, Zhenzhen Lu, Ruixue Zhu

**Affiliations:** ^1^ Department of Emergency Intensive Care Unit, The Affiliated Hospital of Qingdao University, Qingdao, China; ^2^ Clinical Medicine of Zhengzhou University, Zhengzhou, China; ^3^ Department of Health Management, The Affiliated Hospital of Qingdao University, Qingdao, China

**Keywords:** gut microbiota, metformin, sepsis, sepsis-associated acute lung injury (SALI), aged rats

## Abstract

**Background:**

Recent studies reported the association between the changes in gut microbiota and sepsis, but there is unclear for the gut microbes on aged sepsis is associated acute lung injury (SALI), and metformin treatment for the change in gut microbiota. This study aimed to investigate the effect of metformin on gut microbiota and SALI in aged rats with sepsis. It also explored the therapeutic mechanism and the effect of metformin on aged rats with SALI.

**Methods:**

Aged 20-21 months SD rats were categorized into three groups: sham-operated rats (AgS group), rats with cecal ligation and puncture (CLP)-induced sepsis (AgCLP group), and rats treated with metformin (100 mg/kg) orally 1 h after CLP treatment (AgMET group). We collected feces from rats and analyzed them by 16S rRNA sequencing. Further, the lung samples were collected for histological analysis and quantitative real-time PCR (qPCR) assay and so on.

**Results:**

This study showed that some pathological changes occurring in the lungs of aged rats, such as hemorrhage, edema, and inflammation, improved after metformin treatment; the number of hepatocyte death increased in the AgCLP group, and decreased in the AgMET group. Moreover, metformin relieved SALI inflammation and damage. Importantly, the gut microbiota composition among the three groups in aged SALI rats was different. In particular, the proportion of E. coli and K. pneumoniae was higher in AgCLP group rats than AgS group rats and AgMET group rats; while metformin could increase the proportion of Firmicutes, Lactobacillus, Ruminococcus_1 and Lactobacillus_johnsonii in aged SALI rats. Moreover, Prevotella_9, Klebsiella and Escherichia_Shigella were correlated positively with the inflammatory factor IL-1 in the lung tissues; Firmicutes was correlated negatively with the inflammatory factor IL-1 and IL-6 in the lung tissues.

**Conclusions:**

Our findings suggested that metformin could improve SALI and gut microbiota in aged rats, which could provide a potential therapeutic treatment for SALI in aged sepsis.

## Introduction

Sepsis refers to life-threatening organ dysfunction caused by the dysregulation of the host’s response to infection (Singer et al., 2016), and it’s mortality as high as 33%, which affects millions of people worldwide (Angus et al., 2001; Dellinger, 2003; Buchman et al., 2020). Furthermore, studies showed more than 60% septic patients are elderly people (>65 years), and an exponential increase in the incidence and mortality of sepsis in the elderly patients (Milbrandt et al., 2010; Mankowski et al., 2020). The lung is the first organ involved in the progress of sepsis. According to epidemiological data, about 50% of septic patients complicated with acute lung injury (ALI) or acute respiratory distress syndrome (ARDS) in the intensive care unit(ICU) (Sevransky et al., 2009; Aziz et al., 2018). As high mortality of the sepsis-related lung injury (SALI) patients and a poor prognosis in SALI suggest a lack of clinically feasible therapeutic methods. Therefore, studying the pathogenesis of SALI to search a new effective therapy method is necessary.

The gut microbiota is the the chief regulator in maintaining homeostasis of the host (Honda and Littman, 2016). The gut microbial composition changes with age (Yatsunenko et al., 2012), as such from infant to adult shows from dominant Bififidobacterium to Bacteroidetes and Clostridia genus, and to age shows the Centenarians’ gut microbiota with pathogenic microbiota increasing, which different from the infant and adults (Mariat et al., 2009; Claesson et al., 2012; Rampelli et al., 2013). Furthermore, the above gut microbial composition varies indicates the decreased short-chain fatty acid (SCFA) production, leading to the intestinal inflammation and the mucosa reduction and gut permeability increasing. More importantly, the Centenarians’ gut microbiota, Specifically, the relative abundance of the Faecalibacterium, Eubacteriaceae, Lactobacillus and Clostridia decreased and the relative abundance of the Proteobacteria and Bacilli is reduction, resulting the productive SCFA gut microbiota is decreasing, and yet changed pathogen is increasing in the elderly (Rampelli et al., 2013). Additionally, the previous study showed that chronic mild inflammation in the elderly, which might lead to an abnormal increase in intestinal wall permeability (Buford et al., 2018). Therefore, maintaining the balance of the gut microbiota and improving the gut permeability may be effective measures for treating age-related SALI.

Metformin is the first-line treatment in patients with type 2 diabetes (Flory and Lipska, 2019). Studies confirmed that metformin had an anti-inflammatory effect on the expression of inflammatory factors (Cameron et al., 2016; Wu et al., 2018). Furthermore, some studies showed that metformin delayed aging by reducing the production of reactive oxygen species (Valencia et al., 2017). Metformin is taken orally and absorbed mainly in the small intestine (Buse et al., 2016; Honda and Littman, 2016). Metformin also affects the resident intestinal microbiota and can correct intestinal damage by augmenting the intestinal microbiota (Lee et al., 2018; Maniar et al., 2018; Brandt et al., 2019). This study was performed to investigate the effect of metformin on the intestinal microbiota of senile rats with sepsis and assess whether metformin could be an effective drug for treating SALI.

## Materials and methods

### Animals

Twenty-four male Sprague–Dawley (SD) rats (6–8 weeks) were purchased from Beijing Vital River Laboratory Animal Technology (Beijing, China) and housed until 20–21 months. All rats were housed in a specific pathogen-free animal laboratory. The rats were fed a standard diet and purified water under controlled laboratory conditions (12-h light/dark cycle). They were randomly divided into three groups: sham-operated rats (AgS group, *n*=4), rats with cecal ligation and puncture (CLP)-induced sepsis (AgCLP group, *n*=12), and rats treated with oral metformin (100 mg/kg) 1 h after AgCLP treatment (AgMET group, *n*=8). The AgCLP model was used in this study, and anesthesia was provided by injecting hydantoin (10%, 3–4 mL/g) into the peritoneal cavity. Microsurgery was performed in the midline of the abdomen and involved ligating one half of the free end of the cecum, puncturing the cecum with a 21-gauge needle in two locations at the ligature site, and applying gentle pressure until the feces were extruded. The bowel was then placed back into the abdominal cavity, and the incision was closed. The procedure was performed by the same person to minimize the impact of different ligation and puncture sites on the results. After closing the incision, the rats were injected subcutaneously with normal saline (1 mL/100 g) at 37°C and placed back in the cage to rewarm for 1 h. In the AgS group, the rats underwent only open surgery and did not undergo ligation or puncture of the cecum. The rats in the metformin group were given metformin (25 mg/kg) intragastrically 1 h after the surgery. All rats were sacrificed 24 h after CLP treatment. Only five aged rats with SALI survived in the AgCLP group, and four aged rats with SALI survived in the AgMET group after 24 h of CLP treatment. This animal experiment was approved by the Life Science Ethics Review Committee of Zhengzhou University.

### Histological analysis

The tissues from rat lungs were analyzed using hematoxylin and eosin (H&E) staining. The lung and kidney tissues were fixed in 4% paraformaldehyde for 24h and embedded in paraffin. The tissues were then stained and observed under a light microscope. The degree of alveolar congestion, hemorrhage, infiltration and aggregation of neutrophils or leucocytes, and alveolar wall thickness was observed. The lung sections were scored using the aforementioned indicators, with a maximum score of 16.

### Terminal deoxynucleotide transferase d-UTP nick-end labeling assay

The lung tissues were paraffin-embedded and fixed, and the proportion of apoptotic cells was determined using a terminal deoxynucleotide transferase d-UTP nick-end labeling (TUNEL) assay and fluorescence microscopy.

### 16S rRNA gene sequencing for gut microbiota analysis

First, extraction of genome DNA: we used the CTAB/SDS method to extract the genome DNA, and its’ concentration and purity was monitored on 1% agarose gels. And then we diluted the DNA to 1ng/μL by using sterile water based on the concentration. Next, the primers 341F (5’-CCTAyGGRBGCasCAG-3’) and 806R (5’-GGA CTA CNN GGG TAT CTA AT-3’) were used to amplify the 16S rRNA genes of 16SV3–V4 regions with the barcode. All PCR reactions were carried out with Phusion^®^ High-Fidelity PCR Master Mix (New England Biolabs) is used to performed all PCR reactions. Then, PCR Products quantification and qualification analysis. Mix same volume of 1X loading buffer (contained SYB green) with PCR products and operate electrophoresis on 2% agarose gel for detection. Samples with bright main strip between 400-450bp(16S) and ITS (100-400bp) were chosen for further experiments. PCR products was mixed in equidensity ratios. Then, the Qiagen Gel Extraction Kit was used to purify the mixture PCR products (Qiagen, Germany).Sequencing libraries were generated using TruSeq^®^ DNA PCR-Free Sample Preparation Kit (Illumina, USA) following manufacturer’s recommendations and index codes were added. The library quality was assessed on the Qubit^®^ 2.0 Fluorometer (Thermo Scientific) and Agilent Bioanalyzer 2100 system. At last, the library was sequenced on an Illumina NovaSeq 6000 platform and 250 bp paired-end reads were generated. According to 97% similarity, we used the Usearch (version 11.0.667) with no ambiguous bases to cluster. The Mothur v1.42.1 and the vegan

package in R-package were used to calculated the Alpha diversity and beta diversity, respectively. The PICRUSt2 software package (https://github.com/picrust/picrust2) used to calculate the pathway enrichment.

### Quantification of mRNA using qRT–PCR

We used TRIzol reagent (TaKaRa, Tokyo, Japan) to extract total RNA from the lungs. The concentration and purity of RNA were quantified using ultraviolet spectroscopy. The corresponding cDNA was synthesized by reverse transcription of mRNA using a TaqMan reverse transcription kit (UE, Suzhou, China). All qRT-PCR was used 40 cycles for amplification, and the results were analyzed by the 2^-ΔΔ^CT method. The gene expression was normalized using reduced glyceraldehyde 3-phosphate dehydrogenase (GAPDH) expression. The gene primers were chemokine (C-C motif) ligand 7 (CCL7) forward primer: CTTCTGTGTGTGCTGCTCAAC, reverse primer: CTATGGCCTCCTCAACCCAC; interleukin (IL)-6 forward primer: AGAGACTTCCAGCCAGTTGC, reverse primer: AGTCTCCTCTCCGGACTTGT; CCL3 forward primer: TGCTGTTCTTCTCTGCACCA, reverse primer: CAGGTCCTTTGGGGTCAGC; IL-1β forward primer: GCAACTGTTCCTGAACTCAACT, reverse primer: ATCTTTTGGGGTCCGTCAACT; chemokine (C-X-C motif) ligand 1 (CXCL1) forward primer: CGCTCGCTTCTCTGTGCA, reverse primer: TTCTGAACCATGGGGGCTTC; and GAPDH forward primer: TGTGAACGGATTTGGCCGTA, reverse primer: GATGGTGATGGGTTTCCCGT.

### Statistical analysis

The GraphPad Prism (Version 6.0; GraphPad Software Inc., USA) R-package were used to statistical analyses. The quantitative data was assessed by mean ± standard deviation. The unidirectional or bidirectional analysis of variance (ANOVA) was used for multiple groups, and an unpaired-sample Student *t* test was used for the statistical analysis in two groups. Furthermore, the Mothur v1.42.1 and the vegan package in R-package were used to calculated the Alpha diversity and beta diversity, respectively. The PICRUSt2 software package (https://github.com/picrust/picrust2) used to calculate the pathway enrichment. *P* value <0.05 indicated a statistically significant difference.

## Results

### Metformin alleviated the inflammation and lung injury in aged rats with SALI

**Figure 1:**
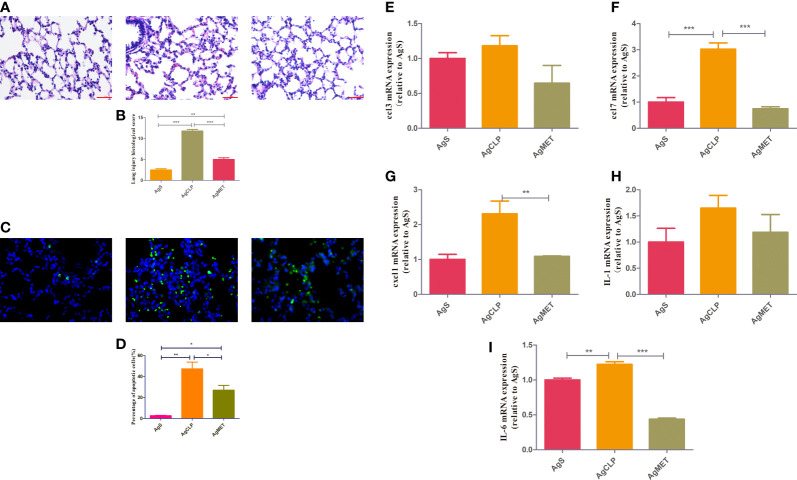
Metformin reduces SALI in aged rats with sepsis. **(A)** H&E staining showed edema and an increased amount of hemorrhage in the lung tissue of rats in the AgCLP group compared with the control group. These changes could be reversed after metformin treatment. **(B)** The level of lung injury was assessed semi-quantitatively based on the lung injury score (*P* < 0.05). **(C)** TUNEL results showed an increased apoptosis in the AgCLP group and a decreased apoptosis in the AgMET group. **(D)** Three sections were taken from each group, and the number of apoptotic cells was measured (*P* < 0.05). **(E-I)** ccl3, ccl7, cxcl1, IL-1 and IL-6 expression increased in AgCLP group and metformin could decreased these factors’ expression. *p<0.05; **p<0.01; ***p<0.001.

A previous study (DeFronzo et al., 2016) reported that metformin may induce kidney failure result of lactic acidosis. Hence, the histological analysis to check whether metformin could induce kidney injury. The results showed that metformin did not cause kidney damage, confirming that the dose of metformin was safe to administer (**Figure S1**). We next performed H&E and TUNEL assays on lung tissues to assess the effect of metformin on SALI in aged rats. The results showed that lung tissue destruction, inflammatory infiltration, and alveolar wall thickening in the aged rats in the AgCLP group compared with the rats in the AgS group. However, lung tissue destruction and inflammatory infiltration were significantly improved in the AgMET group (**Figures 1A, B**, *P* < 0.05). The effect of metformin on apoptotic cells of lung tissues in aged rats with sepsis was determined by assessing the percentage of apoptotic cells through the TUNEL staining. The apoptotic cells of lung tissues significantly increased in aged rats with sepsis compared with that in AgS rats, and metformin treatment attenuated sepsis-induced apoptotic cells of lung tissues (**Figures 1C, D**, *P* < 0.05). Further, the mRNA expression of the inflammatory factors *CCL3*, *CCL7*, *CXCL1*, *IL-1*, and *IL-6* substantially increased in aged rats with SALI compared with the AgS group. However, metformin reversed the expression of these inflammatory factors induced by sepsis (**Figures 1E, I**, *P* < 0.05). Metformin improved the inflammatory response in rats with sepsis, which was consistent with our previously reported results (Liang et al., 2022). Hence, these data suggested that metformin attenuated lung injury and inflammation in aged rats with SALI.

### Effects of metformin on the intestinal microbiota in aged rats

**Figure 2:**
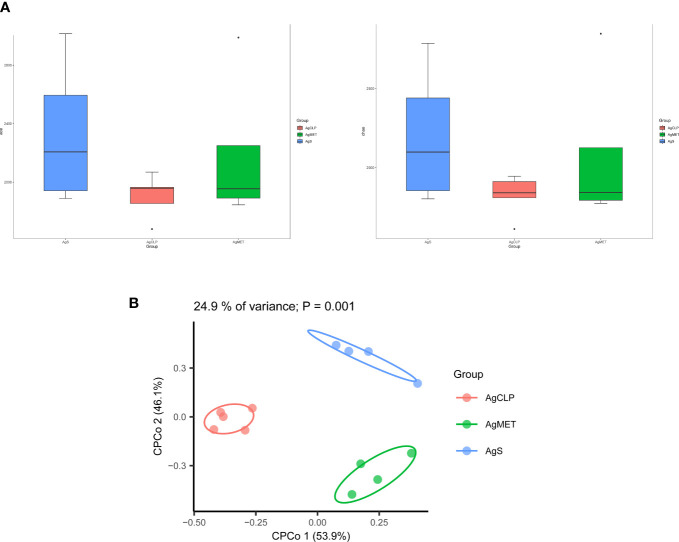
Effect of metformin on intestinal microbiota diversity in aged rats with sepsis. **(A)**16S rRNA metagenomic analysis for α-diversity (ACE, Chao1); **(B)** β-diversity reflected microbial richness in groups and between groups.

16S rRNA metagenomic analysis was used to assess the composition of gut microbiota, evaluating the effect of metformin on gut microbiota in aged rats with SALI. Alpha diversity (ACE, Chao1, Shannon, and Simpson indexes) can reflect the abundance of bacteria in the community. We used ACE and Chao1 to assess the abundance of microbiota. The results showed that metformin could improve the SALI induced the decreased Alpha diversity (**Figure 2A**). Next, we analyzed the β-diversity between bacterial populations. β-diversity assessed the differences of gut microbiota between multiple samples and the changes of microbiome under different factors. The results of β-diversity showed that compared with the AgCLP group, the gut microbiota in AgMET group was similar to that in the AgS group (**Figure 2B**).

**Figure 3:**
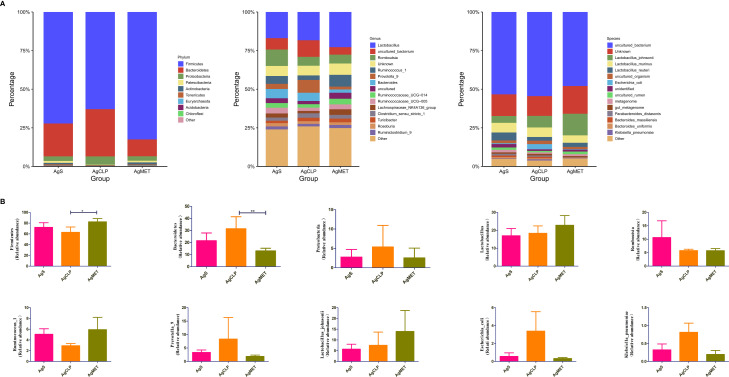
Effects of metformin on intestinal microbiota. **(A)** Three figures show the differences between the microbiota in the AgMET, AgS, and AgCLP groups at the phylum, genus, and species levels, respectively. The abundance of opportunistic pathogenic bacteria increased in the AgCLP group. **(B)** Histogram showing the differential microbiota, highlighting the changes in intestinal microbiota between the experimental groups. *p<0.05; **p<0.01.

**Figure 4:**
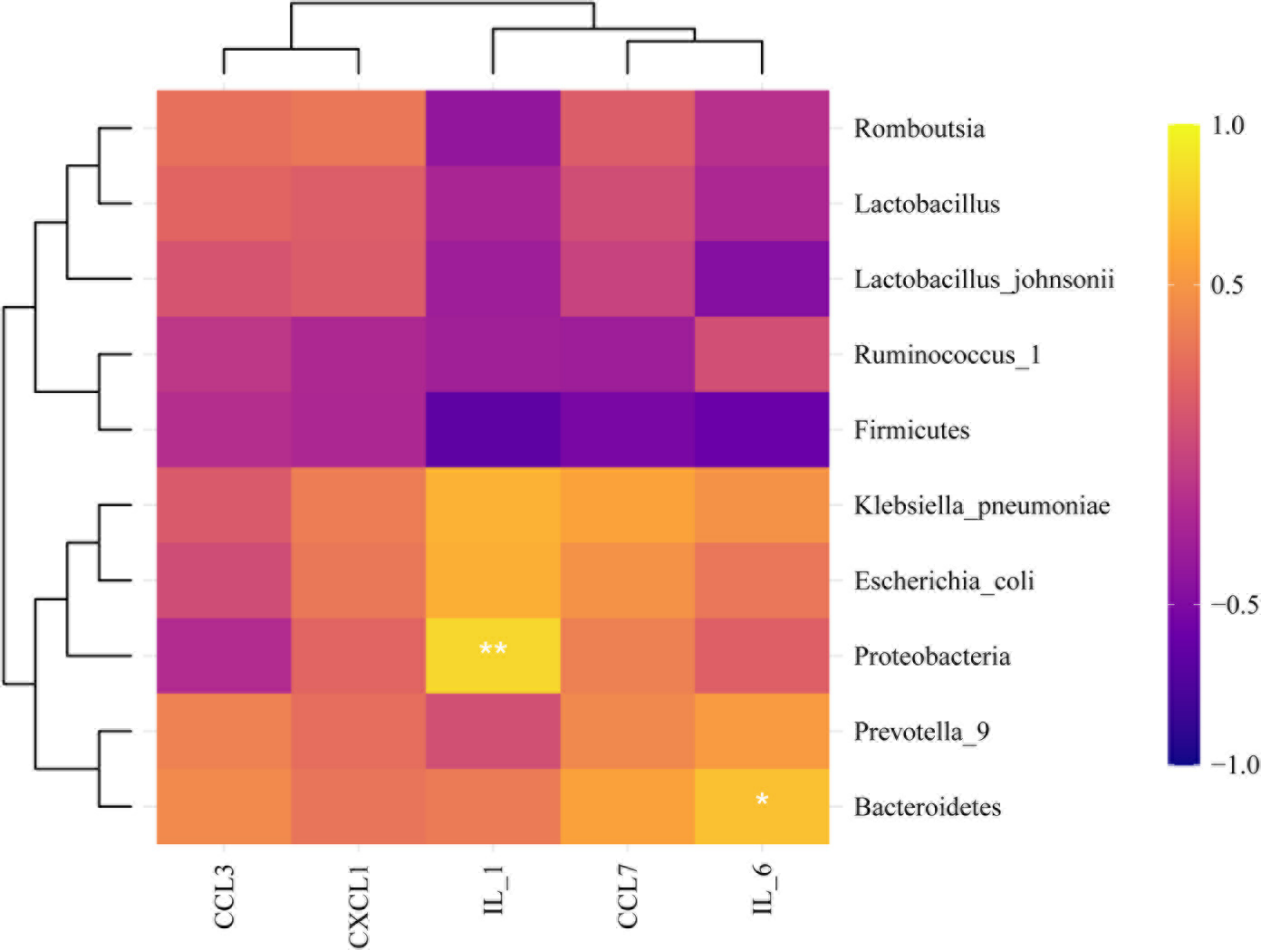
Correlation analysis. Correlation analysis between microbiota and inflammatory parameters.

We explored the effect of metformin on the abundance of gut microbiota. The results showed that compared with the AgS and AgCLP groups, the AgMET group had a higher *Firmicutes*/*Bacteroidetes* ratio (**Figure S2**). At the genus level, the abundance of *Lactobacillus* slightly increased, while the abundance of *Ruminococcus-1* decreased in the AgCLP group compared with the AgS group, and increased after metformin treatment. Furthermore, the increased relative abundance of *Prevotella-9* in the AgCLP group compared with the AgS group, which related with inflammation, while metformin could reverse this change. At the species level, the abundance of *Lactobacillus johnsonii* slightly increased, and *Escherichia coli* and *K. pneumoniae* increased in the AgCLP group compared with the AgS and AgMET groups (**Figures 3A, B**). In the AgCLP group, the increase in the abundance of these opportunistic pathogens increased the inflammatory response of the host, which was consistent with our previous findings (Liang et al., 2022). Furthermore, the abundance of *Romboutsia* and *Ruminococcus-1* decreased and the abundance of *Proteobacteria*, *Escherichia coli*, and *K. pneumoniae* increased in AgCLP group compared with that in the AgMET group, indicating a decrease in the abundance of intestinal microbiota associated with SCFA production. In addition, we analyzed the correlations between inflammatory factors and gut microbiota (**Figure 4**), the results show that *Klebsiella pneumoniae, Escherichia coli, Prevotella-9* and *Proteobacteria* were positively correlated with iL-6, while Romboutsia, Latobacillus, Latobacillus_johnsonii,Firmicutes were negatively correlated with IL-1 and IL-6. Therefore, metformin administration could improve the gut microbiota disorder in aged rats with SALI.

## Discussion

This study assessed the effect of metformin for alleviating inflammation, lung injury and gut microbiota disorder in aged rats with sepsis. Metformin treatment reversed the pathological changes including lung tissue damage, hemorrhage, and edema in aged rats with CLP-induced sepsis. Meanwhile, significant apoptotic cells of lung tissues in the AgCLP group but with a considerable improvement in the AgMET group. The gut microbiota composition in the AgMET group varied from such as an increasing relative abundance of some opportunistic pathogen such as *E. coli* and *K. pneumoniae*, relating with LPS production and inflammation to anti-inflammation, which exerted the protection of aged rats with SALI. These results showed that metformin is a therapeutic alternative for treating aged SALI.

Metformin is a clinical first-line hypoglycemic drug as its specific antihyperglycemic properties with excellent safety profile. Metformin also has other functions such as anti-aging and anti-tumor effects (Shafiee et al., 2014; Pryor et al., 2019). Furthermore, enhanced the lifespan of *Caenorhabditis* by affecting the metabolism of microbial folate and methionine by modifying the gut microbiota (Cabreiro et al., 2013). Metformin could inhibit IκB kinase/nuclear factor-κB activation to suppress the expression of senescence-related factors (Moiseeva et al., 2013). It was later discovered that fecal microbial transplantation decreased the expression of IL-18 (Lee et al., 2019). Metformin improved the intestinal barrier function by modulating the gut microbiota, thereby increasing the number of mucus-producing goblet cells (Hur and Lee, 2015). Additionally, metformin inhibited apoptosis *via* the phosphoinositide 3-kinase/Akt signaling pathway, which was found to be effective in brain injury caused by sepsis (Tang et al., 2017). These studies also supported our findings.

The *Firmicutes* and *Bacteroidetes* is the main gut microbiota in human, accounting for 75.9% and 10.83%, respectively (Pan et al., 2018), which was consistent with the results of this study. We found that the abundance of *Firmicutes* decreased in the AgCLP group compared with the AgS group, and this change was reversed after metformin treatment. *Firmicutes* are mainly Gram-negative bacteria, consisting of specialized anaerobes or parthenogenic anaerobes, of which *Faecalibacterium* is involved in the formation of butyric acid, while *Dialister* is engaged in the final phase of propionic acid production (Nava et al., 2011; Tanca et al., 2017). The abundance of *Firmicutes* decreased noticeably in type 2 diabetes. In this study, AgCLP caused variations in the abundance of intestinal microbiota, such as a decline in the abundance of lactic acid–producing bacteria and probiotics and an increase in the abundance of opportunistic pathogenic bacteria associated with inflammation, such as *E. coli* and *K. pneumoniae*. Metformin treatment reversed these changes, resulting in an increase in the abundance of *L. johnsonii* and a decline in the abundance of *E. coli* and *K. pneumoniae*. *Bacteroides thetaiotaomicron* and *L. johnsonii* reduced the infiltration of intestinal inflammatory cells, alleviated edema, disrupted the cell wall mannans of *Candida albicans*, and inhibited the development of *C. albicans (* Charlet et al., 2020). In sepsis, intestinal barrier dysfunction and increased permeability contribute to the pathological transfer of intestinal bacteria or endotoxins, worsening sepsis (Hassoun et al., 2001; Meng et al., 2017). Metformin further enhances intestinal barrier function by increasing the number of villi through the modulation of intestinal microbiota, such as *Firmicutes* and lactic acid–producing bacteria. *Prevotella* interacts with the immune system and enhances mucosal inflammation mediated by TH17, stimulating epithelial cells to produce inflammatory factors such as IL-8 and IL-6 (Zeng et al., 2019), which agreed with our results.

This study had some limitations. First, the sample size in the experimental groups was small, and this findings should be confirmed by other similar studies, in the other hand, in the next study, we could be further verify the findings. Second, we did not include the metformin-alone group, reducing the rigor of the study. However, after metformin treatment, the kidney injury had no difference between AgCLP group and AgMET group, which suggested the dose of metformin is safe. More importantly, our previous study (Liang et al., 2022) search the effect of metformin alone group on septic aged rats.

## Conclusions

This study indicated that metformin could relieve inflammation, lung injury and gut microbiota in aged rats with SALI. More importantly, metformin reversed the imbalance of gut microbiota such as as increasing relative abundance of opportunistic pathogen such as *E. coli* and *K. pneumoniae*. in aged rats with sepsis, which could provide a potential treatment for aged SALI.

## Data availability statement

The datasets presented in this study can be found in online repositories. The names of the repository/repositories and accession number(s) can be found below: NCBI, PRJNA938428.

## Ethics statement

The animal study was reviewed and approved by the Life Science Ethics Review Committee of the Affiliated Hospital of Qingdao University.

## Author contributions

RZ and YW designed the study. SW, YN, BD, YL and ZL performed the experiments and collected the animal sample, and they also conducted the data analysis. YW wrote the manuscript. All authors contributed to the article and approved the submitted version.

## Supplementary material

Supplementary Figure 1 Metformin alleviated sepsis-associated acute kidney injury.

Supplementary Figure 2
*Firmicutes*/*Bacteroidetes* ratio (*P* < 0.05).
