# Supplementary figures and images for: Effect of metformin on sepsis-associated acute lung injury and gut microbiota in aged rats with sepsis
Source: Front Cell Infect Microbiol. 2023 Mar 9;13:1139436. doi: 10.3389/fcimb.2023.1139436 (PMC10034768; doi:10.3389/fcimb.2023.1139436)

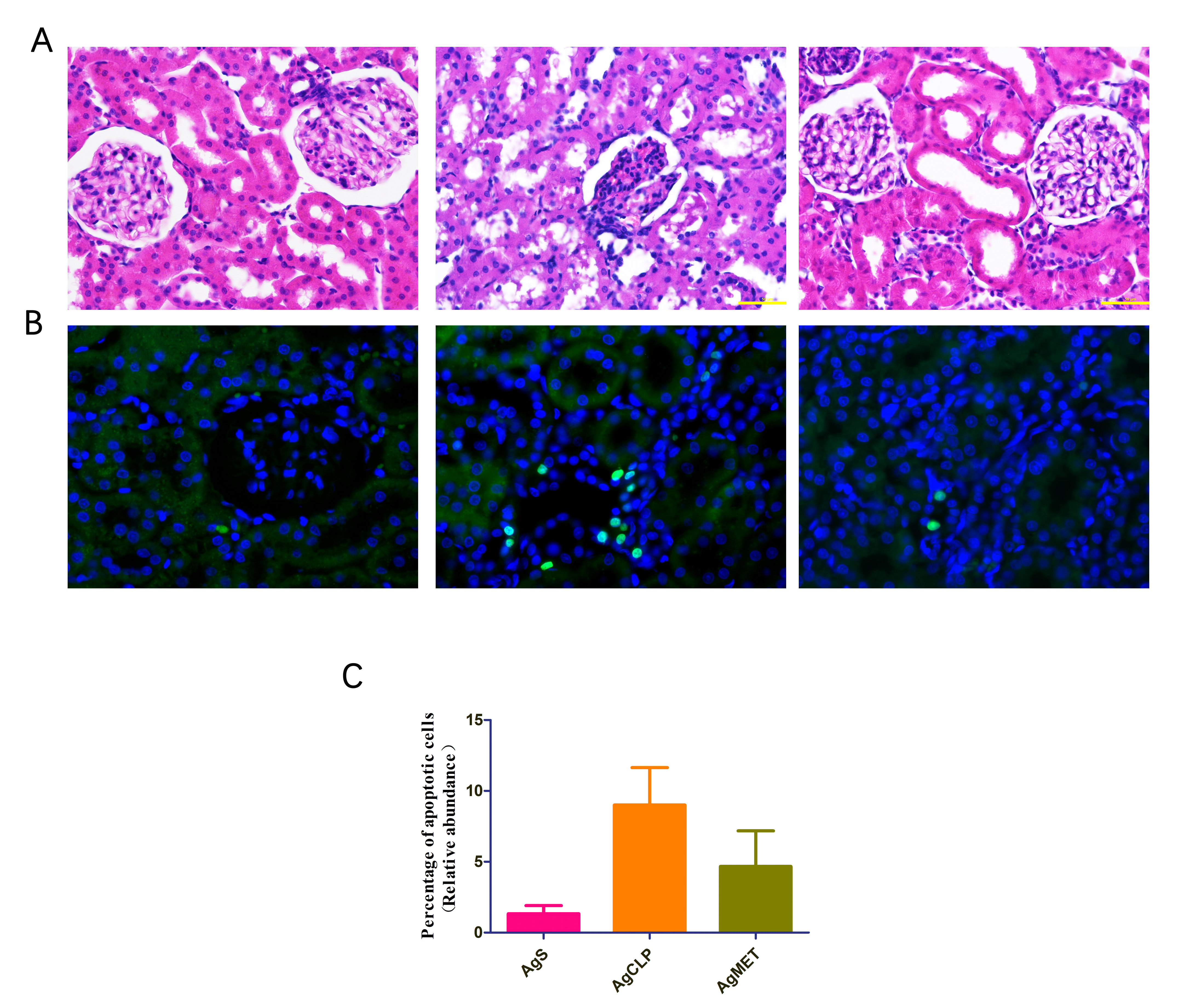

Supplement: Supplementary Figure 1 — Metformin alleviated sepsis-associated acute kidney injury. [file Image_1.tif]
